# L-Arginine abrogates maternal and pre-pubertal codeine exposure-induced impaired spermatogenesis and sperm quality by modulating the levels of mRNA encoding spermatogenic genes

**DOI:** 10.3389/fendo.2023.1180085

**Published:** 2023-07-17

**Authors:** Roland Eghoghosoa Akhigbe, Oladele A. Afolabi, Ayodeji Folorusho Ajayi

**Affiliations:** ^1^ Department of Physiology, Ladoke Akintola University of Technology, Ogbomoso, Oyo State, Nigeria; ^2^ Reproductive Biology and Toxicology Research Laboratory, Oasis of Grace Hospital, Osogbo, Osun State, Nigeria

**Keywords:** arginine, codeine, developmental programming, endocrine disrupting chemicals, infertility, opioid, reproductive toxicity, spermatogenesis

## Abstract

**Introduction:**

Although, codeine has been demonstrated to lower sperm quality; the effects of maternal and prepubertal codeine exposure on male offspring is yet to be reported. In addition, the effect of arginine on codeine-induced decline in sperm quality has not been explored. This study investigated the impact of maternal and prepubertal codeine exposure on spermatogenesis and sperm quality in F1 male Wistar rats to study the effect that codeine may have during recreational use in humans. Also, the effect of arginine supplementation on codeine-induced alteration in spermatogenesis and sperm quality was evaluated.

**Methods:**

Female rats were treated with either 0.5 ml distilled water or codeine orally for eight weeks, and then mated with male rats (female:male, 2:1). The F1 male offsprings of both cohorts were weaned at 3 weeks old and administered distilled water, codeine, arginine, or codeine with arginine orally for eight weeks.

**Results:**

Prepubertal codeine exposure in rats whose dams (female parents) were exposed to codeine delayed puberty and reduced the weight at puberty. Prepubertal codeine exposure exacerbated maternal codeine exposure-induced reduced total and daily spermatid production, sperm count, sperm motility, and normal sperm form, as well as impaired sperm plasma membrane integrity and increased not intact acrosome and damaged sperm DNA integrity. These perturbations were accompanied by a decrease in mRNA levels encoding spermatogenic genes, testicular testosterone and androgen receptor (AR) concentrations, and upregulation of sperm 8-hydroxydeoxyguanosine (8OHdG). Prepubertal arginine supplementation mitigated codeine-induced alterations.

**Discussion:**

This study provides novel experimental evidence that maternal and prepubertal codeine exposure reprogramed spermatogenesis and sperm quality of male FI generation by decreasing mRNA levels encoding spermatogenic genes and AR via oxidative stress-mediated signaling, which was abrogated by prepubertal arginine supplementation.

## Introduction

Male infertility has become a major public health challenge globally with considerable psychological and social distress (1, 2). It accounts for about 50% of the total cases of infertility and has been attributed to altered spermatogenesis and sperm quality (3, 4).

Spermatogenesis is a cascade of molecular processes that occurs in the seminiferous tubules and involves the proliferation and differentiation of male germ cells via mitosis, meiosis, and spermiogenesis (5). The differentiated spermatogonial that are formed from the differentiation of the self-renewing spermatogonial stem cell are transformed into spermatocytes and then into through meiosis into spermatids (6), which in turn undergo spermiogenesis to produce spermatozoa (5, 7). The differentiated sperm cells are highly specialized cells that must be capable of active motility to get into the female genital tract and penetrate the oocyte (8). Besides motility, other factors that influence sperm quality include sperm count, morphology, plasma membrane integrity, acrosome integrity, and DNA integrity (9). Although spermatogenesis is under precise control by spermatogenic genes, testosterone, and androgen receptors (AR) (5, 10–12), spermatogenesis and sperm quality may be altered by exposure to some drug (13), including codeine (14–18).

Although codeine is an effective analgesic and antitussive, it is widely abused as a recreational drug due to its addictive property and tolerance development within a short period (19). The chronic use of codeine has been reported to cause organ damage via oxidative stress-sensitive signaling (20–22). In addition, codeine has been reported to impair spermatogenesis, induce low sperm quality, and reduce circulating testosterone (14–17) via oxidative stress-mediated downregulation of HER2/Ki67 signaling and upregulation of caspase 3 signaling and p53/Bcl-2 pathway (17, 18). Although apoptosis seems to play a role, codeine primarily impaired spermatogenesis and lowered sperm quality through the induction of sperm cell oxidative DNA damage as depicted by a rise in the sperm 8-hydroxydeoxyguanosine (8OHdG) level (15). Furthermore, codeine has been shown to impair ovarian steroidogenesis and folliculogenesis by inducing oxidative stress, inflammation, and apoptosis (23). Since exposure to drugs, including codeine, does not only influence the health of the individual but can also affect the phenotype of the following generations (2, 14) and codeine abuse is seen in both males and females (14); it is likely that codeine abuse in pregnant females will alter spermatogenesis and sperm quality in male FI offspring. This may be by modulating the spermatogenic genes that control spermatogenesis (10–12) or direct toxicity to the germ cells (2). Also, with the rise in codeine abuse observed across different age groups (14), there is a tendency that individuals birthed by mothers who abuse codeine will abuse codeine.

On the other hand, arginine has been reported to exert antioxidant and anti-inflammatory properties (24) with a likelihood to attenuate the negative effects of codeine, but its role in male reproductive function is understudied. The antioxidant and anti-inflammatory activities of arginine has been linked to its ability to suppress the generation of malondialdehyde (MDA), a marker of lipid peroxidation, and up-regulate the concentration of reduced glutathione (GSH) and the activities of enzymatic antioxidants (24). The intake of arginine has been shown to enhance its concentrations in the plasma and tissue (24) and to promote the biosynthesis of nitric oxide, which is an important vasodilator (25). Although there is a scarcity of data on the effect of arginine on codeine-induced male reproductive dysfunction, our previous study (26) revealed that arginine attenuated codeine-induced delay in puberty attainment and impaired penile and sexual function. This effect of arginine on codeine-exposed rats was demonstrated to be associated with the upregulation of circulating testosterone and androgen receptor in the testis (26). Given these properties of arginine and the knowledge that codeine impairs male fertility via an oxidative stress-dependent pathway, the present study investigated whether or not maternal and prepubertal codeine exposure reprograms spermatogenesis and sperm quality in adult male of F1 generation to study the effect that codeine may have during recreational use in humans, as well as to demonstrate the possible mechanisms involved and the likely role of prepubertal arginine supplementation.

## Methods

### Chemicals and reagents

Codeine was donated by the National Drug Law Enforcement Agency (NDLEA), Nigeria for research purposes. Arginine was commercially procured from Loba Chemie, Mumbai, India. Other reagents used were of analytical grade and obtained from Sigma Chemical Co., USA unless otherwise indicated.

### Animals, treatments, and collection of samples

**Figure 1:**
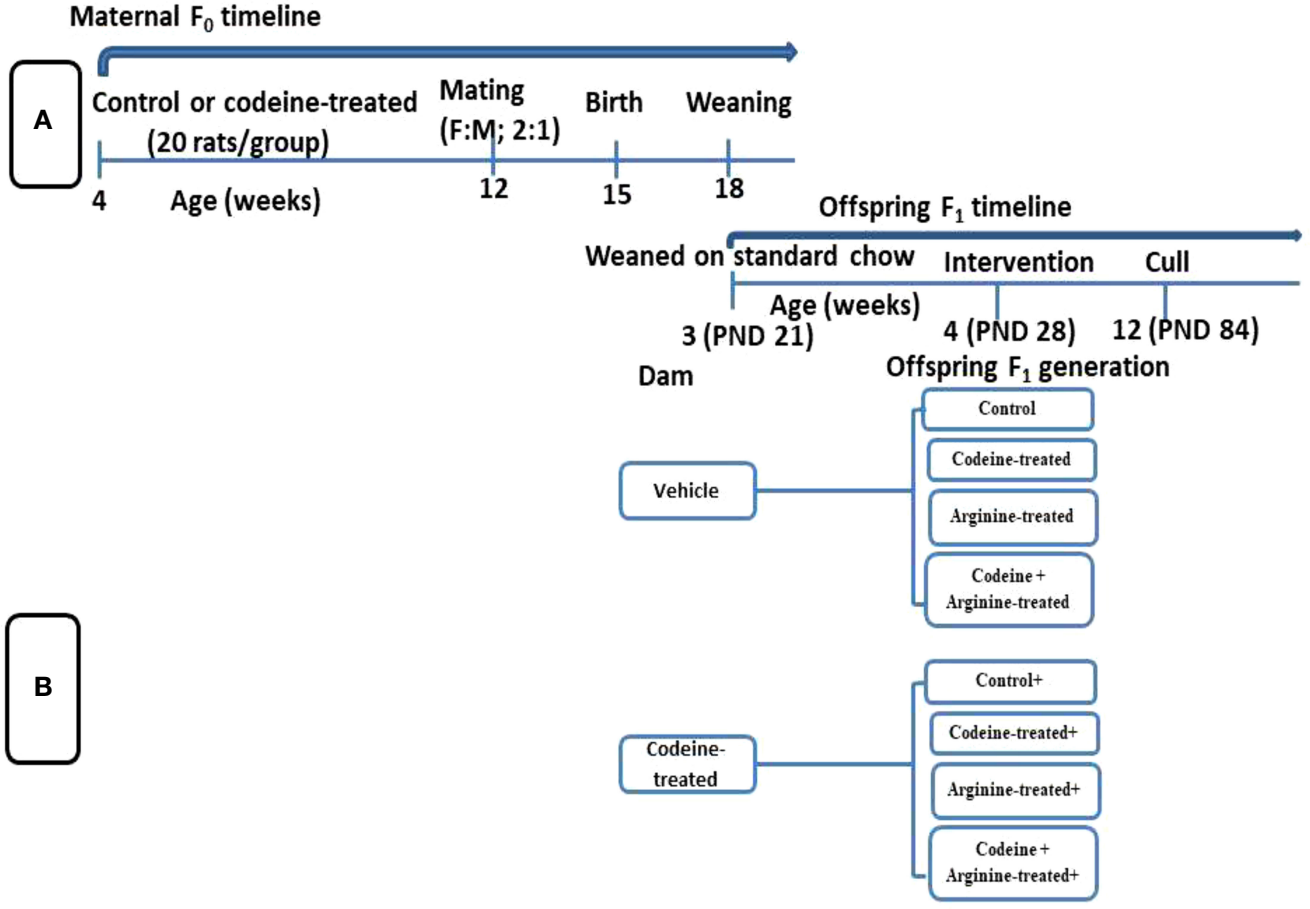
Schematic illustration of the study design showing the maternal timeline **(A)** and offspring timeline **(B)**.

The study protocol and treatment regimens are presented in **Figure 1** and **Table 1**. Forty newly weaned 3-week old female Wistar rats of similar age were randomly selected from twenty dams that had been monitored from the 17th day of gestation to determine the precise date of birth. The newly weaned female rats were housed in well-ventilated plastic cages (2 rats/cage). The rats were allowed to feed on rat chow and drink filtered tap water *ad libitum* and were subjected to the natural light/dark cycle. The rats were humanely handled under the Guide for the Care and Use of Laboratory Animals of the National Academy of Science (NAS) published by the National Institute of Health (NIH). The study was approved by the Ethics Review Committee, Ministry of Health, Oyo State, Nigeria. The study was reported per ARRIVE guidelines.

**Table 1:** Groups and their various treatments.

Group	Label	Treatment		Arginine
		Maternal codeine	Prepubertal codeine	
1	Control	–	–	–
2	Codeine	–	+	–
3	Arginine	–	–	+
4	Codeine+Arginine	–	+	+
5	Control+	+	–	–
6	Codeine+	+	+	–
7	Arginine+	+	–	+
8	Codeine+Arginine+	+	+	+

-, Did not receive; +, Received.

Rats were randomly assigned to either the control that received 0.5 ml of distilled water or the codeine-treated that received 5 mg/kg of codeine via oral administration for eight weeks. Administration commenced at 4 weeks old. Afterward, the female rats were mated with male Wistar rat that were maintained on rat chow only and not exposed to codeine (2:1; female: male per cage) for seven days. To eliminate the effect of parity on the study, all dams were virgins at the time of mating and only their first litters were used for the study. Mating was confirmed by the presence of sperm cells on the vaginal smear, and pregnancy was confirmed by body weight gain a week after mating. Rats were maintained on their pre-conception treatments throughout pregnancy and lactation.

All F1 generation pups were delivered within 3 days. The pups were left undisturbed for the first week of life to prevent post-natal stress. At 3 weeks old (weaning), forty male offsprings (F1 generation) of comparable weights from each cohort (control and codeine-treated cohorts) were randomly allocated into four equal groups each; the control, codeine-treated (5 mg/kg of codeine), arginine-treated (300 mg/kg of arginine), and codeine + arginine-treated. Administration commenced a week post-weaning and lasted for eight weeks. The administration was via gavage. The dose of codeine used is the submaximal peak dose obtained from the dose-response curve of our pilot study as previously reported (18, 26, 27) and that of arginine was as previously reported (28). Animals were culled at 12 weeks via euthanasia using intraperitoneal ketamine (4 mg/kg bw) and xylazine (40 mg/kg bw). Blood samples were obtained via cardiac puncture and the testes were excised and trimmed off of surrounding tissues. The absolute paired testicular weight was obtained as the sum of the weight of the right and left testes, while the relative testicular weight was obtained as the percentage of the paired testicular weight divided by the body weight. Five of the left testes were randomly selected for histopathological evaluation, while the remaining five were used for total and daily spermatid production evaluation. Also, five of the right testes were homogenized in cold phosphate buffer solution for biochemical parameters, while the remaining five were homogenized in TRIzol reagent for the assay of mRNA levels of spermatogenic genes.

### Age and weight at puberty

The onset of puberty was determined by preputial membrane separation (29), which was monitored starting from postnatal day 24 until it was observed. The age and weight (using a sensitive electronic weighing scale) of the animals at the onset of puberty were determined and recorded.

### Spermatid production and sperm quality

Total and daily spermatid production was evaluated by counting the spermatids resistant to sonication as earlier reported (30, 31). Briefly, the testicular tunica albuginea was removed and the parenchyma was homogenized in 5 ml of 0.5% saline-triton by sonication for 30 minutes, 12 kHz. The obtained samples were diluted in saline (1: 10) and the spermatids were counted using a hemocytometer. The total spermatid production (TSP) was determined as the number of spermatid per testis and per gram of testis. The daily spermatid production was calculated by dividing TSP by 6.1 days.

To determine the sperm parameters, the left caudal epididymides were incised and the content (sperm cells) was carefully expressed into a clean Petri dish containing 2.5 ml of Biggers-Whitten-Whittingham capacitation medium, and the sperm motility, count, and morphology were immediately evaluated as previously documented (27, 32). A drop of the epididymal sperm suspension was introduced onto a clean glass slide and covered with a coverslip. About 200 sperm cells per replicate were viewed in 5 different fields at ×200 and ×400 magnifications under a light microscope to evaluate sperm motility. To evaluate the sperm count, epididymal sperm suspension was mixed with a solution of 50 g of sodium bicarbonate and 10 ml of 35% (v/v) formalin in 1000 ml of purified water. About 10 μL of the sperm solution was then transferred into each chamber of the Improved Neubauer Haemocytometer using a Pasteur pipette. At least 200 sperm cells were counted per replicate at ×200 and ×400 magnification under light microscopy. To assess sperm morphology, a smear of the epididymal sperm suspension was made on a slide, air-dried, fixed, and stained with Eosin. About 200 sperm cells were examined per replicate for various defects in sperm form.

Eosin-nigrosin staining and hypo-osmotic swelling test (HOST) were used to determine sperm plasma membrane integrity (3, 33). For the eosin-nigrosin staining, 5 μL of the epididymal sperm suspension and 5 μL of the staining solution containing 1% eosin Y and 10% nigrosin were mixed, pipetted onto a clean sterile slide, smeared with another slide, and air-dried at room temperature. Two hundred spermatozoa were viewed per slide by light microscopy at ×1000 magnification and classified as either intact/viable plasma membrane if unstained or not intact/non-viable plasma membrane if stained. Sperm cells with intact plasma membrane were determined as those with intact membrane integrity. For the HOST assay, 0.1 mL of the epididymal sperm suspension was added to 1 mL of hypo-osmotic solution (containing 0.735 g of sodium citrate dehydrate and 1.351 g of fructose in 100 mL of distilled water). The sperm suspension was gently mixed by drawing the sample in and out of the pipette and then incubated at 37°C for 30 and 60 min. After incubation, a drop of the mixture was placed on a clean sterile slide and viewed under light microscopy at ×400 magnification. Two hundred sperm cells per replicate were counted. Sperm cells with swollen curl-up tail tips were considered as the sperm cells with intact plasma membrane and regarded as HOST-positive/reactive, while those without swollen tails were considered as the sperm cells without intact plasma membrane and regarded as HOST-negative.

Acrosome integrity was determined as previously documented (33, 34). About 5 µL of each epididymal sperm suspension was incubated with 5 µL of the single-stain solution containing 1% (w/v) Rose Bengal, 1% (w/v) fast green FCF and 40% ethanol in 200 mM disodium phosphate buffer containing 100 mM citric acid at pH 7.2 for 1 minute at 37°C. About 10 µL of the solution was then pipetted onto a slide and a smear was made, air-dried at 37°C, and analyzed by light microscopy at ×1000. Two hundred sperm cells were counted per slide and classified as intact or not intact acrosome according to the staining density. Intact acrosomes stained purple-blue and exhibited a conical shape, while not intact acrosomes (absent or reacted acrosomes) appeared as blunt and colorless edges.

Sperm DNA integrity was evaluated following Toluidine blue staining (3, 35). The dried smear of the epididymal sperm suspension was fixed with freshly-made 96% ethanol: acetone (1:1) at 4°C for 30 min and air-dried. The dried smear was then hydrolyzed with 0.1 N HCl at 4°C for 5 min and rinsed three times in distilled water for 2 min per rinse. The slide was then stained with Toluidine blue, allowed to dry at room temperature for 5 min, then viewed under light microscopy at ×1000 magnification. Two hundred spermatozoa per replicate were evaluated, and the percentage of stained sperm heads was calculated. Sperm heads stained light blue or blue were considered those with DNA of intact integrity while those stained dark blue were considered those with damaged DNA.

### mRNA levels of spermatogenic genes

The levels of mRNA that encode *Ndrg4, Kit*, *Rhcg*, *Lrrc34, and Lgals1* genes were determined as earlier reported (36). The harvested testes were homogenized in TRIzol reagent (Inqaba Biotech, South Africa). The homogenate was partitioned into three phases by the addition of chloroform and then centrifuged for 10 minutes. The upper layer was carefully pipetted into a clean tube. Isoamyl alcohol, the precipitating medium, was added to the solution containing the RNA pellets and vortex for 30 minutes. The supernatant was decanted to obtain the RNA pellet. Further precipitation and cleaning of the RNA pellet were done by adding 70% ethanol. DNase was used to treat the sample to remove DNA contamination and obtain a DNase-free RNA. The RNA was converted to cDNA using ProtoScriptFirst Strand cDNA Synthesis Kit (NEB). *Ndrg4, Kit*, *Rhcg*, *Lrrc34, and Lgals1* mRNA genes were amplified by Reverse Transcriptase Polymerase Chain Reaction (RT-PCR) using the primer sets in **Supplementary Table 1**. The PCR amplicon was submitted for a densitometric run in agarose 2% gel electrophoresis using a TBE buffer solution (Bio Concept, Switzerland). Snapshots revealing the relative density of the DNA bands were taken under blue light documentation (Bluebox, USA). Image J Software was used to quantify the intensities of the bands.

### Testicular testosterone and androgen receptor concentrations

Serum levels of luteinizing hormone (LH), follicle-stimulating hormone (FSH) and testosterone were assayed using ELISA kit (Monobind Inc., USA) per the manufacturers’ guidelines. Testicular testosterone (Monobind Inc., USA) and androgen receptor concentrations (Biorbyt Ltd., Cambridge, CB4 OWY) were determined using an ELISA kit and following the manufacturers’ guidelines.

### Sperm oxidative DNA damage

The concentration of 8OHdG in the epididymal sperm suspension was employed as an index of sperm oxidative DNA damage (15) using an ELISA kit (Elabscience Biotechnology Co., Ltd, USA) per the manufacturer’s guidelines.

### Histopathological analysis

Samples of the testicular tissues were preserved in Bouin’s solution and dehydrated in ascending grades of alcohol. About 4 to 5 µm thick section was obtained and stained with hematoxylin-eosin stain. The sections were viewed under a light microscope (Omax 40x-2, 500x LED binocular Lab compound Microscope, M82EZ-C5OS, China) and images were taken at ×100 magnification. The germ cells were manually counted. The spermatogonia and Sertoli cells lie on the basal membrane, while the primary spermatocytes, secondary spermatocytes and spermatids lie in the adluminal compartment, between the basement membrane and the lumen. The Sertoli cells are oval-shaped with pale nuclei, while the spermatogonia are rounded with pale nuclei. The primary spermatocytes have heterochromatic nuclei, while the secondary spermatocytes lie between the primary spermatocytes and the round spermatids. Germ cell count was determined by two experts and the mean for each was determined as the value.

### Statistical analysis

Graph Pad Prism (Graph Pad Software Inc., La Jolla, CA, USA) was employed to analyze the obtained data. Comparisons were made using one-way analysis of variance (ANOVA) and Tukey’s posthoc test. Multiple correlational studies were conducted to determine the association between spermatogenic genes and spermatogenic cell count. Results are expressed as mean± standard deviation. *P* values less than 0.05 were considered statistically significant.

## Results

### Age and weight at puberty onset

**Figure 2:**
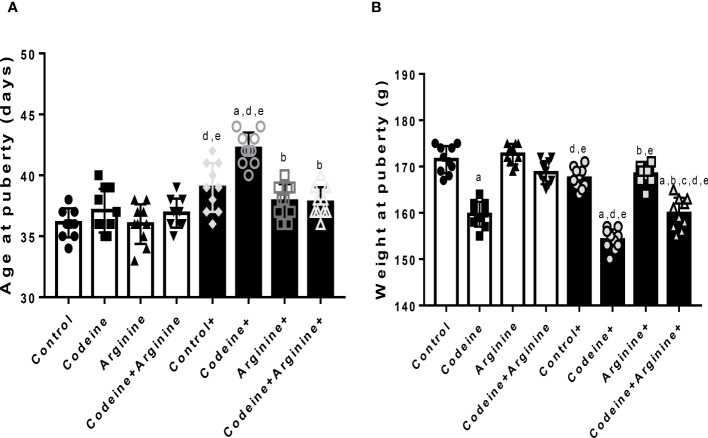
Effect of maternal and prepubertal codeine use, and prepubertal arginine use on age **(A)** and weight **(B)** at puberty in F1 male offsprings. ^+^Offsprings of dams exposed to codeine. Data are expressed as mean ± SD for ten rats per group and analyzed by one-way analysis of variance (ANOVA) followed by Tukey’s posthoc test for pair-wise comparison. ^a^ *P* < 0.05 vs control of same cohort, ^b^ *P* < 0.05 vs codeine of same cohort,. ^c^ *P* < 0.05 vs arginine of same cohort, ^d^ *P* < 0.05 vs control without maternal codeine exposure, ^e^ *P* < 0.05 vs same prepubertal treatment without maternal codeine exposure.

The onset of puberty was determined by preputial membrane separation. Although prepubertal codeine exposure did not significantly alter the age of puberty onset of F1 male offsprings (36.1 ± 1.19 vs. 37.1 ± 1.79, *P=*0.8069), it reduced the weight at puberty (171.5 ± 2.95 vs. 159.7 ± 2.66, *P<*0.0001) (**Figure 2**). Also, maternal codeine exposure significantly delayed puberty (36.1 ± 1.19 vs. 39.00 ± 2.01, *P=*0.0011) and reduced the weight at puberty (171.50 ± 2.95 vs. 167.50 ± 2.27, *P=*0.0181) when compared with the control. Prepubertal codeine exposure in offsprings whose dams were exposed to codeine further significantly delayed puberty (167.50 ± 2.27 vs. 154.20 ± 2.25, *P=*0.0002) and reduced the weight at puberty (65.40 ± 2.31 vs. 52.60 ± 2.06, *P<*0.0001) when compared to the control in the same cohort. However, arginine, when administered alone or in combination with codeine, prevented codeine-induced delayed puberty (42.20 ± 1.31 vs. 37.90 ± 1.37, *P<*0.001; 42.20 ± 1.31 vs. 37.80 ± 1.22, *P<*0.001 respectively) and reduction in weight at puberty (154.20 ± 2.25 vs. 168.40 ± 2.01, *P<*0.001; 154.20 ± 2.25 vs. 159.90 ± 3.38, *P=*0.001 respectively).

### Absolute and relative testicular weight

Prepubertal codeine exposure significantly reduced absolute paired testicular weight when compared with the control (2.83 ± 0.06 vs. 1.53 ± 0.04, *P<*0.0001) (**Table 2**). Arginine, when administered alone (2.94 ± 0.07 vs. 2.83 ± 0.06, *P=*0.001, compared with control) and in combination with codeine (2.52 ± 0.04 vs. 1.53 ± 0.03, *P<*0.0001, compared with codeine) significantly improved testicular weight. Furthermore, when compared with the control whose dams were not exposed to codeine, maternal codeine exposure significantly reduced testicular weight (2.83 ± 0.06 vs. 1.67 ± 0.04, *P<*0.0001). Prepubertal codeine exposure worsened maternal codeine exposure-induced reduction in testicular weight (1.67 ± 0.04 vs. 1.25 ± 0.03, *P<*0.0001). Prepubertal arginine supplementation significantly improved maternal codeine exposure-induced reduction in testicular weight (1.67 ± 0.04 vs. 2.69 ± 0.03, *P<*0.0001) and maternal + prepubertal codeine exposure-induced reduction in testicular weight (1.25 ± 0.03 vs. 2.16 ± 0.04, *P<*0.0001). More so, prepubertal codeine exposure significantly reduced relative testicular weight when compared with the control (1.49 ± 0.02 vs. 0.81 ± 0.01, *P<*0.0001). Co-administration of codeine with arginine prevented prepubertal codeine exposure-induced reduction in relative testicular weight (0.81 ± 0.01 vs. 1.33 ± 0.03, *P<*0.001). In addition, when compared with the control whose dams were not exposed to codeine, maternal codeine exposure significantly reduced relative testicular weight (1.49 ± 0.02 vs. 0.86 ± 0.01, *P<*0.0001). Prepubertal codeine exposure worsened maternal codeine exposure-induced reduction in relative testicular weight (0.86 ± 0.02 vs. 0.65 ± 0.02, *P<*0.0001). Prepubertal arginine supplementation significantly improved maternal codeine exposure-induced reduction in relative testicular weight (1.41 ± 0.03 vs. 0.86 ± 0.02, *P<* 0.0001) and maternal + prepubertal codeine exposure-induced reduction in relative testicular weight (1.41 ± 0.03 vs. 0.65 ± 0.03, *P<* 0.0001).

**Table 2:** Effect of maternal and prepubertal codeine use, and prepubertal arginine use on absolute and relative testicular weight in F1 male offsprings.

	Absolute paired testicular weight (g)	Relative testicular weight (%)
**Control**	2.83 ± 0.06	1.49 ± 0.02
**Codeine**	1.53 ± 0.03^a^	0.80 ± 0.01^a^
**Arginine**	2.94 ± 0.06^a,b^	1.56 ± 0.04^b^
**Codeine + Arginine**	2.52 ± 0.04^a,b,c^	1.33 ± 0.02^a,b,c^
**Control^+^**	1.67 ± 0.04^d^	0.86 ± 0.01^d^
**Codeine^+^**	1.25 ± 0.03^a,d,e^	0.65 ± 0.02^a,d,e^
**Arginine^+^**	2.69 ± 0.03^a,b,d,e^	1.41 ± 0.02^a,b,d,e^
**Codeine + Arginine^+^**	2.16 ± 0.04^a,b,d,e^	1.12 ± 0.03^a,b,c,d,e^

^+^Offsprings of dams exposed to codeine.

Data are expressed as mean ± SD for five rats per group and analyzed by one-way analysis of variance (ANOVA) followed by Tukey’s posthoc test for pair-wise comparison.

^a^P < 0.05 vs control of same cohort, ^b^P < 0.05 vs codeine of same cohort, ^c^P < 0.05 vs arginine of same cohort, ^d^P < 0.05 vs control without maternal codeine exposure, ^e^P < 0.05 vs same prepubertal treatment without maternal codeine exposure.

### Spermatid production and sperm characterization

**Figure 3:**
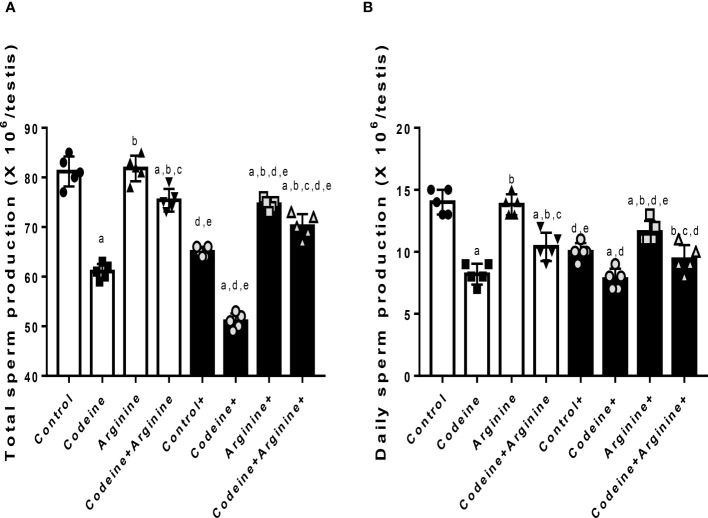
Effect of maternal and prepubertal codeine use, and prepubertal arginine use on total **(A)** and daily **(B)** spermatid production in F1 male offsprings. ^+^Offsprings of dams exposed to codeine. Data are expressed as mean ± SD for five rats per group and analyzed by one-way analysis of variance (ANOVA) followed by Tukey’s posthoc test for pair-wise comparison. ^a^ *P* < 0.05 vs control of same cohort, ^b^ *P* < 0.05 vs codeine of same cohort, ^c^ *P* < 0.05 vs arginine of same cohort, ^d^ *P* < 0.05 vs control without maternal codeine exposure, ^e^ *P* < 0.05 vs same prepubertal treatment without maternal codeine exposure.

Prepubertal codeine exposure significantly reduced total (80.90 ± 2.72 vs. 60.70 ± 1.94, *P*<0.0001) and daily (13.70 ± 1.05 vs. 8.10 ± 0.87, *P<*0.0001) spermatid production when compared with the control (**Figure 3**). Arginine, when administered alone, did not significantly alter total (80.90 ± 2.72 vs. 81.10 ± 2.47, *P*>0.9999) and daily (13.70 ± 1.05 vs. 13.70 ± 0.94, *P*>0.9999) spermatid production when compared with the control; but when administered in combination with codeine, significantly restored total (60.70 ± 1.94 vs. 75.20 ± 2.30, *P*<0.0001) and daily (8.10 ± 0.87 vs. 10.30 ± 0.94, *P*<0.0001) spermatid production when compared with the codeine-treated animals; although, spermatid production was not fully restored by arginine. Furthermore, when compared with the control whose dams were not exposed to codeine, maternal codeine exposure significantly reduced total and daily spermatid production (80.90 ± 2.72 vs. 65.40 ± 2.32, *P<*0.0001; 13.70 ± 1.05 vs. 9.70 ± 0.94, *P<*0.0001 respectively). These observations were worsened by prepubertal codeine exposure in rats whose dams were exposed to codeine (65.40 ± 2.31 vs. 52.60 ± 2.06, *P<*0.0001; 9.70 ± 0.94 vs. 8.00 ± 0.81, *P=*0.0031 for total and daily spermatid production respectively). However, prepubertal arginine supplementation blunted maternal codeine exposure-induced reduction in total spermatid production (65.40 ± 2.31 vs. 73.90 ± 1.66, *P<*0.0001) and daily spermatid production (9.70 ± 0.94 vs. 11.80 ± 0.78, *P<*0.0001) as well as maternal and prepubertal codeine exposure-induced reduction in total spermatid production (52.60 ± 2.06 vs. 70.70 ± 1.94, *P<*0.0001) and daily spermatid production (8.00 ± 0.81 vs. 9.50 ± 1.08, *P=*0.0139).

In addition, prepubertal codeine exposure significantly reduced sperm count (9.64 ± 0.22 vs. 7.57 ± 0.27, *P<*0.0001), sperm motility (89.20 ± 2.61 vs. 65.70 ± 1.94, *P<*0.0001), and increased abnormal sperm morphology (8.60 ± 1.77 vs. 22.70 ± 1.41, *P<*0.0001) when compared with the control (**Table 3**). Although when compared with the control, arginine did not significantly alter sperm count (9.64 ± 0.22 vs. 9.71 ± 0.27, *P=* 0.9971), sperm motility (89.20 ± 2.61 vs. 89.50 ± 2.46, *P*>0.9999), and sperm morphology (8.60 ± 1.77 vs. 7.20 ± 1.47, *P=*0.5191); when administered with codeine, arginine significantly reversed prepubertal codeine-induced alterations in sperm count (7.57 ± 0.27 vs. 8.69 ± 0.16, *P<*0.0001), sperm motility (65.70 ± 1.94 vs. 77.60 ± 2.11, *P<*0.0001), and sperm morphology (22.70 ± 1.41 vs. 13.20 ± 1.61, *P<*0.0001). Moreover, maternal codeine exposure caused a reduction in sperm count (9.64 ± 0.22 vs. 8.13 ± 0.17, *P<*0.0001), sperm motility (89.20 ± 2.61 vs. 78.40 ± 2.22, *P<*0.0001), and a rise in abnormal sperm morphology (8.60 ± 1.77 vs. 13.00 ± 1.70, *P<*0.0001) when compared with the control male rats whose dams were not exposed to codeine. Prepubertal codeine exposure in rats whose dams were exposed to codeine led to a significant reduction in sperm count (8.13 ± 0.17 vs. 7.00 ± 0.20, *P<*0.0001), sperm motility (78.40 ± 2.22 vs. 55.50 ± 1.58, *P<* 0.0001), and a rise in abnormal sperm morphology (13.00 ± 1.70, 29.50 ± 1.78, *P<* 0.0001) when compared with the control male rats without prepubertal codeine exposure but whose dams were exposed to codeine orally. Prepubertal arginine supplementation in rats whose dams were exposed to codeine improved maternal codeine exposure-led alterations in sperm count (8.13 ± 0.17 vs. 8.68 ± 0.20, *P<*0.0001) and abnormal sperm morphology (13.00 ± 1.70 vs. 9.99 ± 1.19, *P=* 0.0012), but did not significantly improve sperm motility (78.40 ± 2.22 vs. 79.30 ± 3.30, *P=*0.9905). However, when compared with rats who had maternal and prepubertal codeine exposure, co-administration of arginine in rats with maternal and prepubertal codeine exposure significantly abrogated codeine-induced reduction in sperm count (7.89 ± 0.26 vs. 8.68 ± 0.20, *P<*0.0001), sperm motility (55.50 ± 1.58 vs. 67.80 ± 2.65, *P<*0.0001), and increase in abnormal sperm morphology (29.50 ± 1.78 vs. 13.80 ± 1.75, *P<*0.0001).

**Table 3:** Effect of maternal and prepubertal codeine use, and prepubertal arginine use on epididymal sperm characterization in F1 male offsprings.

	Sperm count (x 10^6^ per ml)	Sperm motility (%)	Abnormal sperm morphology (%)
**Control**	9.64 ± 0.22	89.20 ± 2.61	8.60 ± 1.77
**Codeine**	7.57 ± 0.28^a^	65.70 ± 1.94^a^	22.71 ± 1.42^a^
**Arginine**	9.71 ± 0.27^b^	89.50 ± 2.46^b^	7.20 ± 1.47^b^
**Codeine + Arginine**	8.69 ± 0.16^a,b,c^	77.60 ± 2.12^a,b,c^	13.20 ± 1.6^a,b,c^
**Control^+^**	8.13 ± 0.17^d,e^	78.40 ± 2.22^d,e^	13.00 ± 1.70^d,e^
**Codeine^+^**	7.00 ± 0.20^a,d,e^	55.50 ± 1.58^a,d,e^	29.50 ± 1.78^a,d,e^
**Arginine^+^**	8.68 ± 0.21^a,b,d,e^	79.30 ± 3.30^b,d,e^	9.90 ± 1.19^a,b,e^
**Codeine + Arginine^+^**	7.89 ± 0.26^b,c,d,e^	67.80 ± 2.65^a,b,c,d,e^	13.80 ± 1.75^b,c,d^

^+^Offsprings of dams exposed to codeine.

Data are expressed as mean ± SD for five rats per group and analyzed by one-way analysis of variance (ANOVA) followed by Tukey’s posthoc test for pair-wise comparison.

^a^P < 0.05 vs control of same cohort, ^b^P < 0.05 vs codeine of same cohort, ^c^P < 0.05 vs arginine of same cohort, ^d^P < 0.05 vs control without maternal codeine exposure, ^e^P < 0.05 vs same prepubertal treatment without maternal codeine exposure.

### Sperm membrane integrity

Sperm plasma membrane integrity was determined by the eosin-nigrosin staining technique and hypo-osmotic swelling (HOS) test (HOST) as shown in **Table 4**. When compared with the control, prepubertal exposure to codeine led to a significant reduction in sperm plasma membrane integrity (91.60 ± 2.27 vs. 69.00 ± 1.94, *P<*0.0001 for eosin-nigrosin stain; 72.90 ± 1.66 vs. 52.60 ± 1.71, *P<*0.0001 for HOST at 30 minutes; 66.40 ± 3.13 vs. 44.50 ± 3.34. *P<*0.0001 for HOST at 60 minutes). Also, prepubertal codeine exposure in rats whose dams were exposed to codeine caused a decline in sperm plasma membrane integrity compared to the animals without prepubertal codeine exposure but whose dams were exposed to codeine (75.00 ± 19.81 vs. 59.10 ± 2.02, *P=*0.002 for eosin-nigrosin stain; 65.60 ± 2.11 vs. 43.70 ± 1.63, *P<*0.0001 for HOST at 30 minutes; 60.30 ± 2.00 vs. 39.20 ± 1.61, *P<*0.0001 for HOST at 60 minutes). Prepubertal arginine supplementation significantly prevented the prepubertal codeine-induced reduction in sperm plasma membrane integrity when compared with prepubertal codeine-treated rats (69.00 ± 1.94 vs. 81.10 ± 1.72, *P=* 0.0098 for eosin-nigrosin stain; 52.60 ± 1.71 vs. 62.50 ± 2.27, *P<*0.0001 for HOST at 30 minutes; 44.50 ± 3.34 vs. 57.10 ± 1.85, *P<*0.0001 for HOST at 60 minutes), and maternal and prepubertal codeine exposure-induced decline in sperm plasma membrane integrity (59.10 ± 2.02 vs. 71.00 ± 2.05, *P=*0.0118 for eosin-nigrosin stain; 43.70 ± 1.63 vs. 60.90 ± 2.33, *P<*0.0001 for HOST at 30 minutes; 39.20 ± 1.61 vs. 56.50 ± 2.22, *P<*0.0001 for HOST at 60 minutes). However, prepubertal arginine supplementation did not significantly improve maternal codeine exposure-led reduction in sperm plasma membrane integrity (75.00 ± 19.81 vs. 82.20 ± 3.04, *P=*0.3675 for eosin-nigrosin stain; 65.60 ± 2.11 vs. 65.60 ± 1.50. *P*>0.9999 for HOST at 30 minutes; 60.30 ± 2.00 vs. 61.20 ± 1.61, *P*=0.9863 for HOST at 60 minutes) when compared with control without prepubertal codeine exposure but whose dams were exposed to codeine.

**Table 4:** Effect of maternal and prepubertal codeine use, and prepubertal arginine use on sperm plasma membrane integrity in F1 male offsprings.

	Sperm vitality (Eosin-nigrosin staining) (%)	HOS Test (%)	
		At 30 minutes	At 60 minutes
**Control**	91.60 ± 2.27	72.90 ± 1.66	66.40 ± 3.13
**Codeine**	69.00 ± 1.94^a^	52.60 ± 1.71^a^	44.50 ± 3.34^a^
**Arginine**	92.00 ± 2.94^b^	73.90 ± 1.85^b^	69.40 ± 1.57^a,b^
**Codeine + Arginine**	81.10 ± 1.73^a,b,c^	62.50 ± 2.27^a,b,c^	57.10 ± 1.85^a,b,c^
**Control^+^**	75.05 ± 1.90^d,e^	65.60 ± 2.11^d,e^	60.30 ± 2.00
**Codeine^+^**	59.10 ± 2.02^a,d^	43.70 ± 1.64^a,d,e^	39.20 ± 1.61^a,d^
**Arginine^+^**	82.20 ± 3.04^b^	65.60 ± 1.51^b,d,e^	61.20 ± 1.61^b,e^
**Codeine + Arginine^+^**	71.00 ± 2.05^b,c,d^	60.90 ± 2.33^a,b,c,d^	56.50 ± 2.22^a,b,c,d^

^+^Offsprings of dams exposed to codeine.

Data are expressed as mean ± SD for five rats per group and analyzed by one-way analysis of variance (ANOVA) followed by Tukey’s posthoc test for pair-wise comparison.

^a^P < 0.05 vs control of same cohort, ^b^P < 0.05 vs codeine of same cohort, ^c^P < 0.05 vs arginine of same cohort, ^d^P < 0.05 vs control without maternal codeine exposure, ^e^P < 0.05 vs same prepubertal treatment without maternal codeine exposure.

### Germ cell count

Although spermatogonial and Sertoli cell counts were comparable across the groups, maternal and maternal with prepubertal codeine exposure significantly reduced primary spermatocytes, secondary spermatocytes, and spermatid counts (**Table 5**). The reduction in these germ cells was prevented, although not completely, by arginine supplementation.

**Table 5:** Effect of maternal and prepubertal codeine use, and prepubertal arginine use on germ cell and Sertoli cell counts in F1 male offsprings.

	Spermatogonia (n)	Primary spermatocytes (n)	Secondary spermatocytes (n)	Spermatids (n)	Sertoli cells (n)
**Control**	62.40 ± 1.67	42.67 ± 1.52	37.33 ± 1.52	50.33 ± 1.53	17.33 ± 0.57
**Codeine**	58.80 ± 1.48	25.67 ± 1.53^a^	17.33 ± 1.53^a^	41.33 ± 0.57^a^	16.33 ± 1.51
**Arginine**	60.20 ± 2.58	42.33 ± 1.53^b^	37.67 ± 0.57^b^	51.33 ± 1.51^b^	18.00 ± 1.00
**Codeine + Arginine**	58.40 ± 1.67	41.00 ± 2.00^b^	37.00 ± 1.00^b^	49.33 ± 1.52^b^	17.00 ± 1.02
**Control^+^**	59.40 ± 2.07	34.33 ± 1.52^d,e^	25.33 ± 1.54^d,e^	50.00 ± 1.00	16.00 ± 1.01
**Codeine^+^**	58.60 ± 1.14	19.33 ± 1.55^d,e^	14.00 ± 1.00^a,d^	40.00 ± 1.01^a,d^	16.67 ± 0.58
**Arginine^+^**	60.80 ± 2.86	35.00 ± 2.00^b,d,e^	28.67 ± 1.53^a,d,e^	49.00 ± 1.01^b^	16.67 ± 0.56
**Codeine + Arginine^+^**	59.40 ± 2.07	35.00 ± 1.00^b,d,e^	27.67 ± 0.57^a,d,e^	47.67 ± 1.15^b^	15.67 ± 0.57

^+^Offsprings of dams exposed to codeine.

Data are expressed as mean ± SD for five rats per group and analyzed by one-way analysis of variance (ANOVA) followed by Tukey’s posthoc test for pair-wise comparison.

^a^P < 0.05 vs control of same cohort, ^b^P < 0.05 vs codeine of same cohort, ^c^P < 0.05 vs arginine of same cohort, ^d^P < 0.05 vs control without maternal codeine exposure, ^e^P < 0.05 vs same prepubertal treatment without maternal codeine exposure.

### mRNA encoding spermatogenic genes in sperm cell subtypes

**Figure 4:**
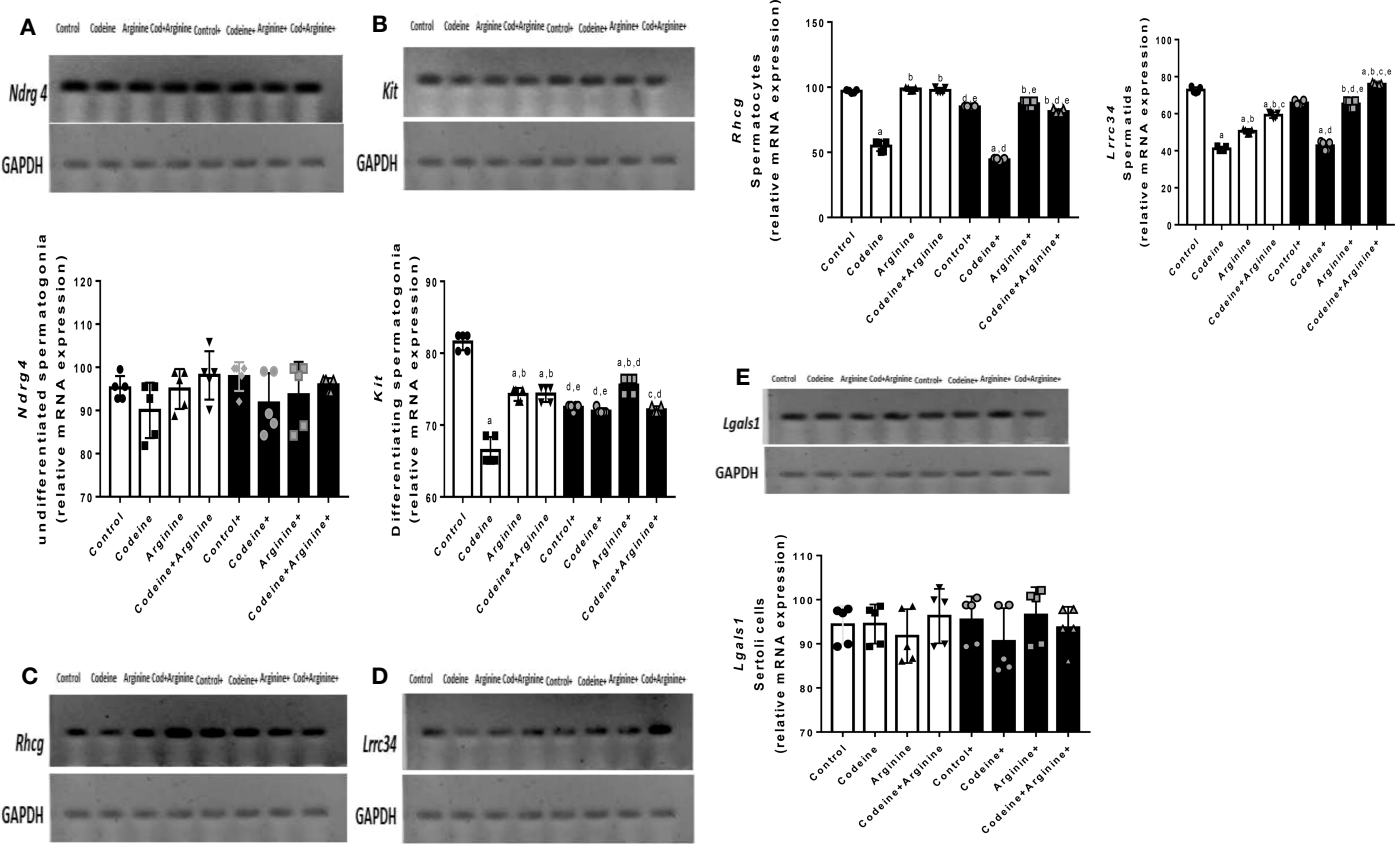
Effect of maternal and prepubertal codeine use, and prepubertal arginine use on the levels of mRNA encoding spermatogenic genes, Ndrg4 **(A)**, Kit **(B)**, Rhcg **(C)**, Lrrc34 **(D)** and Rhcg **(E)** in F1 male offsprings. ^+^Offsprings of dams exposed to codeine. Data are expressed as mean±SD for five rats per group and analyzed by one-way analysis of variance (ANOVA) followed by Tukey’s posthoc test for pair-wise comparison. ^a^ *P* < 0.05 vs control of same cohort, ^b^ *P* < 0.05 vs codeine of same cohort, ^c^ *P* < 0.05 vs arginine of same cohort, ^d^ *P* < 0.05 vs control without maternal codeine exposure, ^e^ *P* < 0.05 vs same prepubertal treatment without maternal codeine exposure.

Although codeine and arginine did not alter the levels of mRNA encoding *Ndrg4 and Lgals1*genes, the levels of mRNA encoding *Kit*, *Rhcg*, and *Lrrc34* genes were significantly affected by codeine and arginine (**Figure 4**). When compared with the control, prepubertal codeine exposure led to a significant reduction in the levels of mRNA encoding *Kit* (81.36 ± 1.22 vs. 66.81 ± 1.96, *P<*0.0001), *Rhcg* (96.71 ± 1.59 vs. 54.24 ± 4.95, *P<*0.0001), and *Lrrc34* (72.95 ± 1.58 vs. 41.32 ± 1.55, *P<*0.0001), which was improved by co-administration of arginine (66.81 ± 1.96 vs. 74.12 ± 1.17, *P<*0.0001 for *Kit*; 54.24 ± 4.95 vs. 97.98 ± 2.78, *P<*0.0001 for *Rhcg*; 41.32 ± 1.55 vs. 58.72 ± 2.04, *P=*0.0004 for *Lrrc34*). Maternal codeine exposure without prepubertal codeine exposure significantly reduced the level of mRNA encoding *Kit* gene when compared with the control (81.36 ± 1.22 vs. 72.43 ± 0.44, *P<*0.0001) and was seemingly not worsened by prepubertal codeine exposure in animals who had prepubertal codeine exposure and whose dams were also exposed to codeine when compared with rats with maternal codeine exposure without prepubertal codeine exposure (72.43 ± 0.44 vs. 71.98 ± 0.44, *P=*0.990). Prepubertal arginine supplementation significantly reversed prepubertal codeine exposure-induced reduction in the level of mRNA encoding *Kit* gene (66.81 ± 1.96 vs. 74.12 ± 1.17, *P=*0.0049) but not maternal and prepubertal codeine exposure-induced reduction in the level of mRNA encoding *Kit* gene (71.98 ± 0.44 vs. 72.20 ± 0.51, *P=* 0.999).

In addition, prepubertal codeine exposure significantly reduced the levels of mRNA encoding *Rhcg* (96.71 ± 1.59 vs. 54.24 ± 4.95, *P<*0.0001) and *Lrrc34* (72.95 ± 1.58 vs. 41.32 ± 1.55, *P<*0.0001) genes when compared with the control. These alterations were significantly improved by arginine supplementation when compared with prepubertal codeine-exposed rats (54.24 ± 4.95 vs. 97.98 ± 2.78, *P<*0.0001 for *Rhcg*; 41.32 ± 1.55 vs. 58.72 ± 2.04, *P=*0.0004 for *Lrrc34*). More so, maternal codeine exposure caused a significant reduction in the level of mRNA that encodes the *Rhcg* gene (96.71 ± 1.59 vs. 84.84 ± 0.52, *P=*0.0251) but did not significantly affect the level of mRNA that encodes the *Lrrc34* gene (72.95 ± 1.58 vs. 66.15 ± 1.67, *P=*0.1061) when compared with the control who had neither maternal nor prepubertal codeine exposure. When compared with animals whose dams were exposed to codeine but did not have prepubertal codeine exposure, prepubertal codeine exposure in rats whose dams were also exposed to codeine showed significant reductions in the levels of mRNA that encode *Rhcg* (84.84 ± 0.52 vs. 44.17 ± 1.19, *P<*0.0001) and the *Lrrc34* (66.15 ± 1.67 vs. 42.20 ± 2.96, *P<*0.0001) genes. Although prepubertal arginine supplementation did not significantly improve maternal codeine exposure-induced reductions in the levels of mRNA that encode *Rhcg* (84.84 ± 0.52 vs. 86.84 ± 3.35, *P=*0.9911) and *Lrrc34* (66.15 ± 1.67 vs. 64.83 ± 3.03, *P=*0.9965) gene, its co-administration with codeine significantly inhibited the maternal and prepubertal codeine-induced decrease in the levels of mRNA that encode *Rhcg* (44.17 ± 1.19 vs. 81.67 ± 2.33, *P<*0.0001) and *Lrrc34* (42.20 ± 2.96 vs. 75.72 ± 0.52, *P<*0.0001) genes.

### Serum levels of male sex hormones, intratesticular testosterone and testicular androgen receptor concentrations

**Figure 5:**
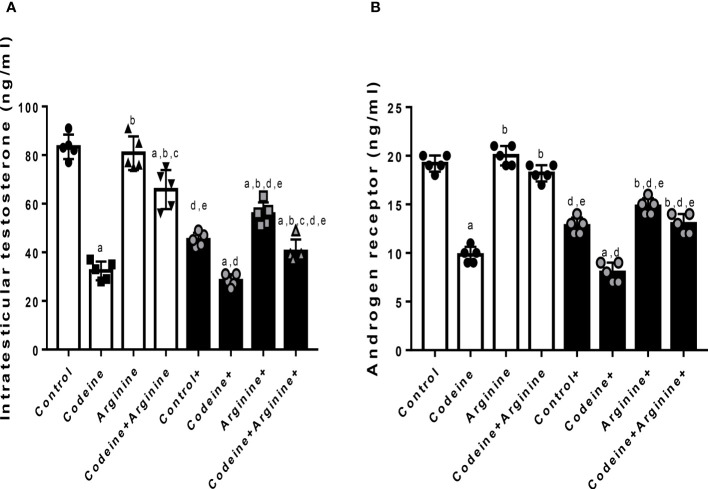
Effect of maternal and prepubertal codeine use, and prepubertal arginine use on intratesticular testosterone **(A)** and androgen receptor **(B)** concentrations in F1 male offsprings. ^+^ Offsprings of dams exposed to codeine. Data are expressed as mean ± SD for five rats per group and analyzed by one-way analysis of variance (ANOVA) followed by Tukey’s posthoc test for pair-wise comparison. ^a^ *P* < 0.05 vs control of same cohort, ^b^ *P* < 0.05 vs codeine of same cohort, ^c^ *P* < 0.05 vs arginine of same cohort, ^d^ *P* < 0.05 vs control without maternal codeine exposure, ^e^ *P* < 0.05 vs same prepubertal treatment without maternal codeine exposure.

Prepubertal exposure to codeine significantly reduced intratesticular testosterone level (80.90 ± 6.85 vs. 32.90 ± 3.38, *P<*0.0001) and testicular concentration of AR (19.00 ± 1.00 vs. 10.00 ± 1.00, *P<*0.0001) when compared with the control (**Figure 5**). These alterations were significantly attenuated by arginine supplementation when compared with prepubertal codeine-exposed rats (32.90 ± 3.38 vs. 68.00 ± 7.43, *P<*0.0001 for intratesticular testosterone; 10.00 ± 1.00 vs. 18.00 ± 1.00, *P<*0.0001 for testicular AR concentration). Moreover, maternal codeine exposure caused a significant reduction in intratesticular testosterone concentration (80.90 ± 6.85 vs. 49.40 ± 4.97, *P<*0.0001) and testicular AR concentration (19.00 ± 1.00 vs. 13.00 ± 1.00, *P<*0.0001) when compared with the control that did not have either maternal or prepubertal codeine exposure. When compared with animals whose dams were exposed to codeine but did not have prepubertal codeine exposure, prepubertal codeine exposure in rats whose dams were also exposed to codeine showed significant reductions in intratesticular testosterone level (49.40 ± 4.97 vs. 29.10 ± 3.03, *P<*0.0001) and testicular AR concentration (13.00 ± 1.00 vs. 8.00 ± 1.00, *P=*0.0003). Although prepubertal arginine supplementation significantly improved maternal codeine exposure-induced reduction in the intratesticular level of testosterone (49.40 ± 4.97 vs. 57.50 ± 4.37, *P=*0.0217), it did not significantly improve testicular AR concentration (13.00 ± 1.00 vs. 15.00 ± 1.00, *P=*0.2829) when compared with animals whose dams were exposed to codeine but did not have prepubertal codeine exposure. However, co-administration of codeine and arginine significantly improved maternal and prepubertal codeine-induced decrease in intratesticular testosterone level (29.10 ± 3.03 vs. 44.60 ± 5.58, *P<*0.0001) and AR concentration (8.00 ± 1.00 vs. 13.00 ± 1.00, *P=*0.0003).

**Figure 6:**
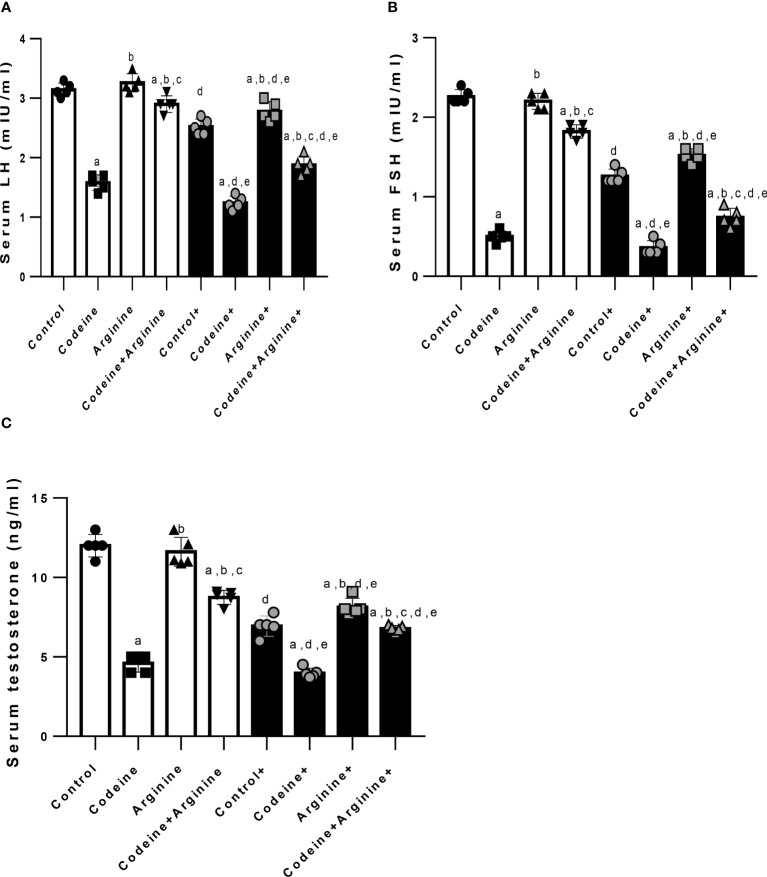
Effect of maternal and prepubertal codeine use, and prepubertal arginine use on serum luteinizing hormone (LH) **(A)**, follicle-stimulating hormone (FSH) **(B)** and testosterone **(C)** levels in F1 male offsprings. ^+^ Offsprings of dams exposed to codeine. Data are expressed as mean ± SD for five rats per group and analyzed by one-way analysis of variance (ANOVA) followed by Tukey’s posthoc test for pair-wise comparison. ^a^ *P* < 0.05 vs control of same cohort, ^b^ *P* < 0.05 vs codeine of same cohort, ^c^ *P* < 0.05 vs arginine of same cohort, ^d^ *P* < 0.05 vs control without maternal codeine exposure, ^e^ *P* < 0.05 vs same prepubertal treatment without maternal codeine exposure.

Prepubertal codeine exposure significantly reduced serum LH, FSH, and testosterone in rats birthed by dams without codeine exposure (p < 0.0001) and those birthed by codeine-exposed dams (p < 0.0001) when compared with the control. Co-administration with arginine attenuated codeine-induced suppression of serum LH, FSH, and testosterone levels in rats birthed by dams with and without codeine exposure (**Figure 6**).

### Acrosome integrity and DNA integrity

**Figure 7:**
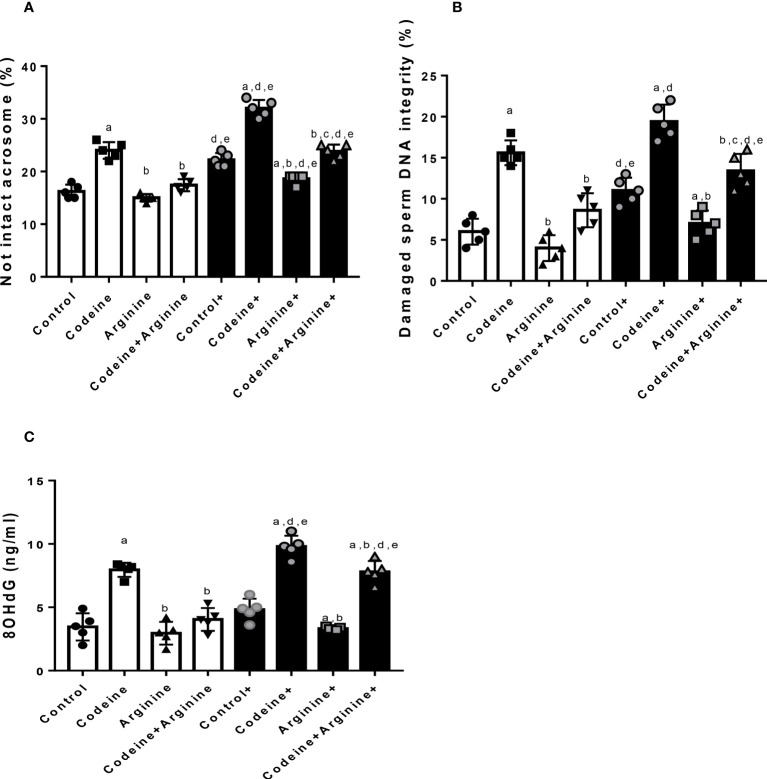
Effect of maternal and prepubertal codeine use, and prepubertal arginine use on sperm acrosome **(A)** DNA integrity **(B)** and oxidative DNA damage **(C)** in F1 male offsprings. ^+^Offsprings of dams exposed to codeine. Data are expressed as mean ± SD for five rats per group and analyzed by one-way analysis of variance (ANOVA) followed by Tukey’s posthoc test for pair-wise comparison. ^a^ *P* < 0.05 vs control of same cohort, ^b^ *P* < 0.05 vs codeine of same cohort, ^c^ *P* < 0.05 vs arginine of same cohort, ^d^ *P* < 0.05 vs control without maternal codeine exposure, ^e^ *P* < 0.05 vs same prepubertal treatment without maternal codeine exposure.

Prepubertal exposure to codeine markedly increased the percentage of not intact acrosome (16.30 ± 1.16 vs. 24.20 ± 1.31, *P<*0.0001), damaged sperm DNA integrity (5.83 ± 1.47 vs. 16.17 ± 1.94, *P<*0.0001), and the concentration of 8-hydroxy-deoxy-guanosine (8OHdG, a marker of oxidative DNA damage) in the sperm suspension (3.46 ± 0.96 vs. 8.20 ± 0.76, *P<*0.0001) when compared with the control (**Figure 7**). These alterations were significantly ameliorated by arginine supplementation when compared with prepubertal codeine-exposed rats (24.20 ± 1.31 vs. 16.90 ± 1.19, *P<*0.0001 for not intact acrosome; 16.17 ± 1.94 vs. 8.50 ± 1.87, *P<*0.0001 for damaged sperm DNA integrity; 8.20 ± 0.76 vs. 4.03 ± 0.80, *P<*0.0001 for 8OHdG level). Besides, maternal codeine exposure caused a significant rise in the percentage of not intact acrosome (16.30 ± 1.16 vs. 22.20 ± 1.22, *P<*0.0001) and damaged sperm DNA integrity (5.83 ± 1.47 vs. 10.83 ± 1.44, *P=*0.0002), but did not considerably alter 8OHdG concentration (3.46 ± 0.96 vs. 4.83 ± 0.77, *P=*0.0913) when compared with the control that did not have either maternal or prepubertal codeine exposure. When compared with animals whose dams were exposed to codeine but did not have prepubertal codeine exposure, prepubertal codeine exposure in rats whose dams were also exposed to codeine showed a significant increase in the percentage of not intact acrosome (22.20 ± 1.22 vs. 32.10 ± 1.44, *P<*0.0001), damaged sperm DNA integrity (10.83 ± 1.44 vs. 19.17 ± 1.94, *P<*0.0001), and 8OHdG concentration (4.83 ± 0.77 vs. 9.80 ± 0.76, *P<*0.0001). In addition, prepubertal arginine supplementation significantly prevented maternal codeine exposure-induced rise in not intact acrosome (22.20 ± 1.22 vs. 18.50 ± 0.97, *P<*0.0001), damaged sperm DNA integrity (10.83 ± 1.47 vs. 6.83 ± 1.47, *P=*0.0052), and 8OHdG concentration (4.83 ± 0.77 vs. 3.33 ± 0.12, *P=*0.0284) when compared with animals whose dams were exposed to codeine but did not have prepubertal codeine exposure. Also, co-administration of codeine and arginine significantly prevented maternal and prepubertal codeine-induced elevation in the percentage of not intact acrosome (32.10 ± 1.44 vs. 23.10 ± 1.45, *P<*0.0001), damaged sperm DNA integrity (19.17 ± 1.94 vs. 13.17 ± 1.92, *P<*0.0001), and 8OHdG concentration (9.80 ± 0.76 vs. 7.80 ± 0.77, *P=*0.0025).

### Histomorphology of the testicular tissues

**Figure 8:**
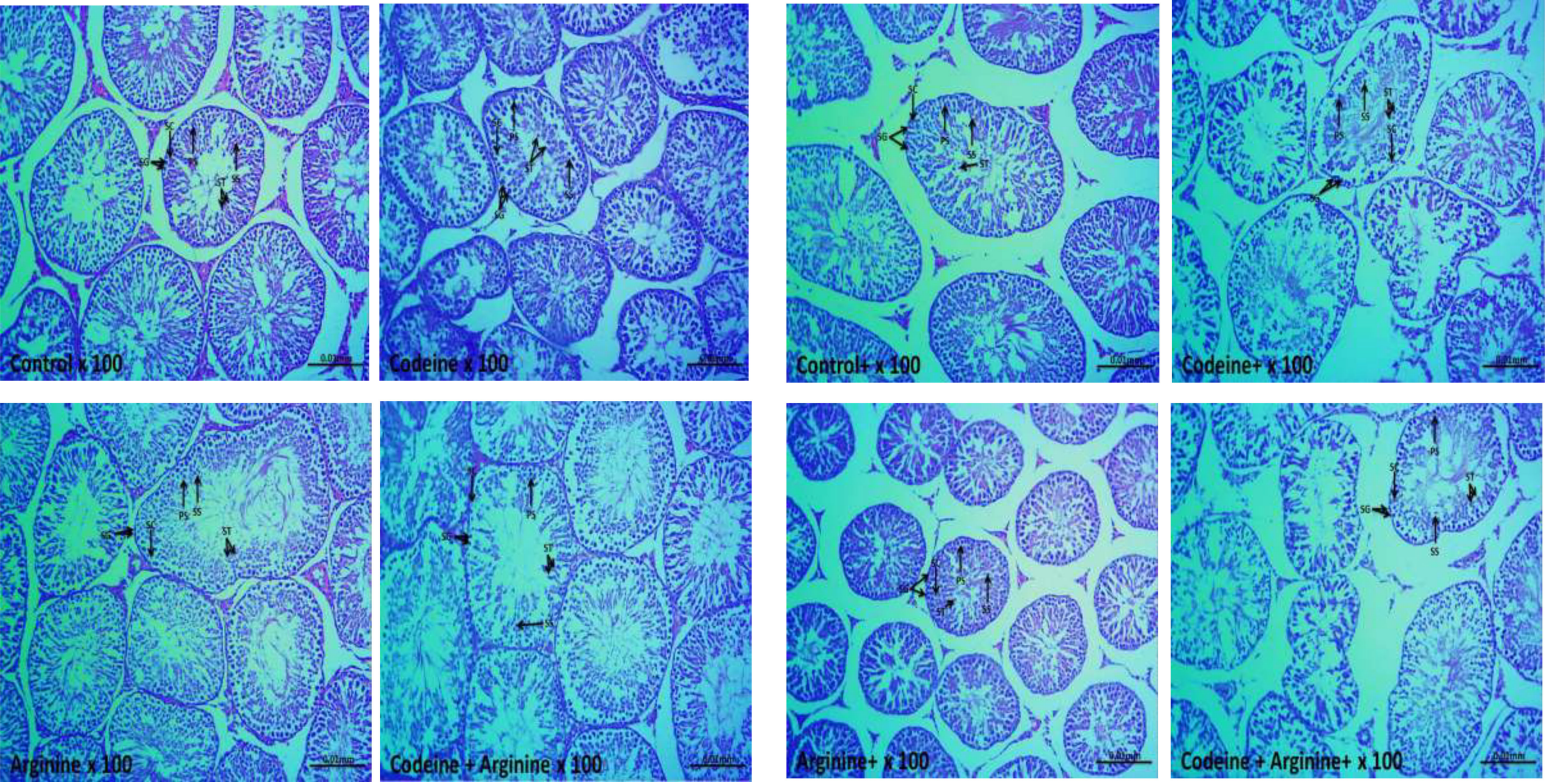
Photomicrographs of the testicular tissues. SG, Spermatogonia; PS, Primary spermatocytes; SS, Secondary spermatocytes; ST, Spermatids; SC, Sertoli cells, ^+^Offsprings of dams exposed to codeine. The control and arginine-treated animals showed preserved testicular histoarchitecture with a thick layer of epithelial cells and tunica propria, normal interstitial space, and normal spermatogenic cells and sperm production. The prepubertal codeine-treated rats showed widened interstitial space, normal spermatogonial and Sertoli cells, but reduced spermatocytes and spermatids. Prepubertal codeine + arginine-treated rats showed prominent restoration of sperm production and improved spermatogenic cells and interstitial space. Control^+^ and arginine^+^ showed normal spermatogenic cells and sperm production with widened interstitial space. The maternal and prepubertal codeine-treated rats showed widened interstitial space, normal spermatogonial and Sertoli cells, but reduced spermatocytes and spermatids. Codeine + Arginine^+^ showed significant restoration of sperm production and improved spermatogenic cells and interstitial space.

The control and arginine-treated animals showed preserved testicular histoarchitecture with a thick layer of epithelial cells and tunica propria, preserved interstitial space and spermatogenic cells and sperm production (**Figure 8**). The prepubertal codeine-treated rats showed widened interstitial space and preserved spermatogonial and Sertoli cells, but reduced spermatocytes and spermatids. Prepubertal codeine + arginine-treated rats showed prominent restoration of sperm production and improved spermatogenic cells and interstitial space. Control^+^ and arginine^+^ showed preserved spermatogenic cells and sperm production with widened interstitial space. The maternal and prepubertal codeine-treated rats showed widened interstitial space and preserved spermatogonial and Sertoli cells, but reduced spermatocytes and spermatids. Codeine + Arginine^+^ showed significant restoration of sperm production and improved spermatogenic cells and interstitial space.

### Correlational studies

Spermatogonial cell count was observed to be positively associated with *Kit*, and *Lrrc34* genes. Primary and secondary cell counts as well as spermatid cell count were noted to be positively associated with *Ndrg*, *Kit*, *Rhcg*, and *Lrrc34* genes (**Table 6**). Findings from these correlational studies infer that spermatogenic cell counts were positively associated with the level of mRNA encoding their genes as well as the upstream and downstream genes.

**Table 6:** Correlation observed between spermatogenic genes and spermatogenic cell count.

	Spermatogonia (*P* value)	Primary spermatocytes *(P* value)	Secondary spermatocytes (*P* value)	Spermatids *(P* value)
***Ndrg***	0.107 (0.510)	0.362 (0.022)*	0.327 (0.039)*	0.366 (0.020)*
***Kit***	0.461 (0.003)*	0.633 (0.000)*	0.664 (0.000)*	0.620 (0.000)*
***Rhcg***	0.292 (0.068)	0.964 (0.000)*	0.949 (0.000)*	0.935 (0.000)*
***Lrrc34***	0.318 (0.046)*	0.601 (0.000)*	0.520 (0.001)*	0.670 (0.000)*
***Lgals1***	0.142 (0.382)	0.159 (0.326)	0.153 (0.346)	0.096 (0.557)

*P < 0.05.

## Discussion

This study provides convincing evidence that maternal and/or prepubertal exposure to codeine impairs spermatogenesis and reduces sperm quality. Also, it demonstrates the positive effects of arginine on the transgenerational effect of codeine. This is seemingly the first evidence provided in the literature on the developmental programming impact of codeine and arginine. Although it was observed that prepubertal codeine exposure did not alter the age and weight of the animals at puberty, it considerably aggravated maternal codeine exposure-induced delayed puberty and reduced weight at puberty. Since reduced body weight is an indicator of substance toxicity (37), this may infer that the toxic effect of codeine is transgenerational. Also, the ability of codeine to delay the onset of puberty confirmed codeine as an endocrine disrupting chemical (38). Arginine supplementation prevented these alterations. This may suggest that arginine militates against the toxic and endocrine disrupting effects of codeine.

In addition, total and daily spermatid production and sperm parameters were profoundly affected by maternal and prepubertal codeine exposures. Spermatogenesis commences at puberty and occurs in the testicular seminiferous tubules under the control of testosterone. Luteinizing hormone (LH) triggers the biosynthesis and release of testosterone by the Leydig cells, while follicle-stimulating hormone (FSH) and testosterone act on the Sertoli cells, which are primarily involved in the regulation of spermatogenesis (15). AR, a nuclear receptor that is expressed in the testicular Leydig cells, Sertoli cells, and peritubular myoid cells, is involved in the maintenance of the blood-testis barrier and the release of sperm cells (16), mediates testosterone-led activation of the mitotic division of spermatogonial; an essential process in the initiation and maintenance of spermatogenesis (15). The present finding that maternal and/or prepubertal codeine reduces the total and daily spermatid production may explain the observed codeine-induced low sperm count. This may be consequent upon the observed reduced spermatogenic cells in codeine-treated animals. This agrees with previous findings that reported low sperm count following codeine exposure (18, 39, 40). Also, the present findings provide an extension of the previous findings. Hence, it is plausible to conclude that codeine does not only reduce sperm count, it also programs spermatogenesis if exposure is during the critical periods of development. Unexpectedly, prepubertal arginine supplementation significantly alleviated maternal and/or prepubertal codeine-induced impaired spermatogenesis. This reveals that arginine may be a potent molecule in reversing the developmental effect of codeine on total and daily spermatid production, probably by its antioxidant effects which may not be unconnected with its ability to promote the biosynthesis of glutathione, a key endogenous antioxidant.

Furthermore, the transcript expressions of specific spermatogenic genes were evaluated to probe the likely role of spermatogenic genes in codeine-induced impairment of spermatogenesis. The mRNA transcript expression of genes selectively expressed in sperm cells subtypes, namely *Ndrg4* (for undifferentiated spermatogonia), *Kit* (for differentiating spermatogonia), *Rhcg* (for spermatocytes), *Lrrc34* (for spermatids), and *Lgals* (for Sertoli cells) (41, 42), were assayed. The finding that prepubertal and/or maternal codeine exposure did not modify mRNA transcript expressions of *Ndrg4* and *Lgals* may indicate that codeine did not significantly alter undifferentiated spermatogonia and Sertoli cell counts respectively. It may also suggest that codeine selectively exerts its effects on sperm cell subtypes since the levels of mRNA that encodes *Kit, Rhcg*, and *Lrrc34* were markedly reduced following codeine exposure. The present finding that codeine decreases the mRNA levels of *Kit, Rhcg*, and *Lrrc34* revealed that codeine reduces the male spermatogenic cells, which are necessary precursors of mature sperm cells. This explains the observed reductions in total and daily spermatid production, primary and secondary spermatocytes, spermatids, and sperm count following codeine exposure. Also, the potency of arginine in inhibiting codeine-induced impaired spermatogenesis may be ascribed to the observed arginine-led upregulation of spermatogenic genes and improvement of the spermatogenic cells.

The toxic effects of EDCs have been established to be mediated by interference with hormone action (43). An important novel finding of this study is the significant reductions in intratesticular testosterone level and AR concentration following prepubertal and/or maternal codeine exposure, which is accompanied by reduced serum LH, FSH and testosterone. It is credible to construe that codeine-induced alteration in testosterone levels and the decrease in AR may directly impair spermatogenesis (35). The present finding that codeine reduces testosterone levels aligns with previous reports that demonstrated that opioids such as tramadol and codeine lower testosterone level (14, 17, 18, 26, 44). Arginine supplementation blocked the codeine-induced decrease in testosterone and AR concentrations possibly by upregulating endocrine signaling pathways, especially the hypothalamic-pituitary-testicular axis evidenced by increased LH and FSH, which culminates in increased testosterone production and release, enhanced AR expression, and consequently improved spermatogenesis and sperm count.

Fertilization is the primary function of the sperm cells and requires preserved sperm plasma membrane and normal morphology, optimal sperm motility, and intact acrosome and sperm DNA (45–47). Findings from this study revealed that prepubertal codeine exposure exaggerated maternal codeine exposure-induced impaired sperm membrane, acrosome integrity, and DNA integrity as well as reduced normal sperm morphology and motility. This finding agrees with previous reports on opioids that demonstrated that opioids like codeine and tramadol reduced sperm motility and negatively affected sperm morphology (15, 16, 18, 44). All these factors combined have a great deal of potential to cause male infertility. Increasing pieces of evidence show that chronic exposure to drugs or abuse of illicit drugs, especially *in utero* and prepubertal that is considered a window of sensitivity for reprotoxic effects and may elicit permanent negative impacts on spermatogenesis later in life (2, 44). The male reproductive system has been reported to be a very sensitive endpoint when the insults ensue during the critical periods of development (45).

The observed codeine-induced reduction in sperm quality may be attributed to the rise in 8OHdG in the sperm suspension following maternal and/or prepubertal codeine exposure. In support of the present data, it has earlier been reported that codeine elicits sperm DNA damage by promoting oxidative stress (15). Hence, it is likely that codeine triggered oxidative sperm damage, leading to increased membrane fluidity, reduced sperm plasma membrane integrity, and a decline in sperm motility (46). Codeine-induced ROS generation may also attack the mitochondrial and nuclear DNA, resulting in impaired acrosome and DNA integrity (47, 48) following oxidative sperm DNA damage. Therefore, the protective role of arginine against codeine-induce spermatogenesis programming is likely connected with its anti-oxidative property (24). Arginine may dampen codeine-induced ROS generation and oxidative injury, promote testosterone production and AR-mediated androgenic action that drives spermatogenesis, and finally enhance total and daily spermatid production, sperm count, and sperm quality.

The observed positive associations between the spermatogonial cell count and *Kit*, and *Lrrc34* genes, and primary and secondary cell counts as well as spermatid cell count and *Ndrg*, *Kit*, *Rhcg*, and *Lrrc34* genes demonstrate that spermatogenic cell counts were positively associated with the level of mRNA encoding their genes as well as the upstream and downstream genes. This may imply that perinatal and prepubertal exposures to codeine reduced spermatogenic cell count by downregulating the levels of mRNA encoding the genes responsible for the differentiation of the cells.

**Figure 9:**
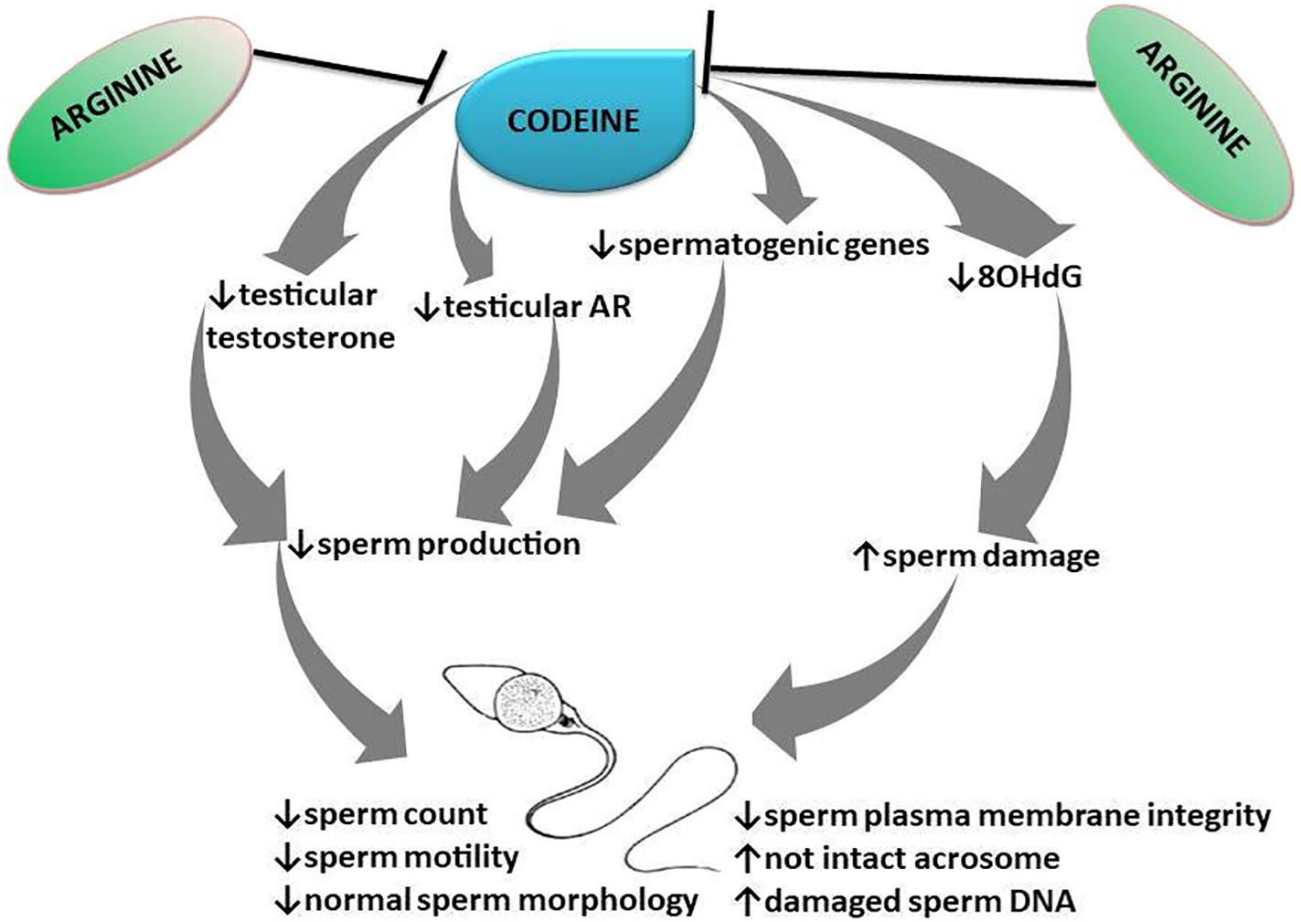
Schematic illustration of the transgenerational effect of codeine and arginine on spermatogenesis and sperm quality.

Collectively, this study provides novel experimental evidence that pre-conception, *in utero*, and prepubertal codeine exposure reprogram spermatogenesis and sperm quality of male FI generation (**Figure 9**). Prepubertal codeine exposure in rats whose dams were exposed to codeine delayed puberty and reduced the weight of animals at puberty. Prepubertal codeine exposure exacerbates maternal codeine exposure-induced impairment of spermatogenesis and sperm quality by decreasing the mRNA levels encoding spermatogenic genes and intratesticular testosterone and AR concentration via the induction of oxidative sperm injury. Prepubertal arginine supplementation exerts a therapeutic and protective effect of maternal codeine exposure-induced and prepubertal codeine exposure-induced decrease in mRNA levels encoding spermatogenic genes and reduced sperm quality, and intratesticular testosterone and AR concentrations by preventing oxidative sperm damage. This study also opens new ground for exploring the possible epigenetic mechanisms of codeine-induced male infertility. More so, the potential therapeutic effect of arginine on testicular developmental toxicity induced by other EDCs should be investigated.

## Data availability statement

The original contributions presented in the study are included in the article/**Supplementary Material**. Further inquiries can be directed to the corresponding authors.

## Ethics statement

The animal study was reviewed and approved by The study was approved by the Ethics Review Committee, Ministry of Health, Oyo State, Nigeria.

## Author contributions

Conceptualization: RA, OA, AA. Data curation: RA. Formal analysis: RA. Investigation: RA. Methodology: RA, OA, AA. Project administration: RA. Resources: RA, OA, AA. Software: RA, OA, AA. Supervision: OA, AA. Validation: OA, AA. Writing – original draft: RA. Writing – review & editing: RA, OA, AA. All authors contributed to the article and approved the submitted version.
