# Supplementary material for: L-Arginine abrogates maternal and pre-pubertal codeine exposure-induced impaired spermatogenesis and sperm quality by modulating the levels of mRNA encoding spermatogenic genes
Source: Front Endocrinol (Lausanne). 2023 Jul 17;14:1180085. doi: 10.3389/fendo.2023.1180085 (PMC10390314; doi:10.3389/fendo.2023.1180085)
Supplement: Supplementary file 1 [file Table_1.docx]

| **Genes** | **Forward primer** | **Reverse primer** | **NCBI Reference Sequence** | **Amplicon size** | **Tm** |
| --- | --- | --- | --- | --- | --- |
| *Ndrg4*  (NDRG family member 4) | GCCCAGCCATCCTTACCTAC | GGCACACCACGAAGTGTTTG | NM_ 001271091.1 | 119 | 58 |
| *Kit*  (KIT proto-oncogene receptor tyrosine kinase) | ATGGAAGATGACGAGCTGGC | AATCTTTGTGATCCGCCCGT | NM_ 022264.1 | 104 | 58 |
| *Rhcg*  (Rh family, C glycoprotein) | AGCGCTGTAGGCTTCAACTT | GTCGGCTTGGATGAGGTTCT | NM_ 183053.1 | 103 | 59 |
| *Lrrc34*  (leucine rich repeat containing 34) | CATCGGTGGTGTGGATGTGA: | GCCTTCAGGTCCAATGTCGT | NM_001044696.1 | 94 | 58 |
| *Lgals1*  (galectin 1) | TACACTTCAACCCCCGCTTC | TGATGCACACCTCCGTGATG | NM_019904.1 | 105 | 59 |

SI Table 1: Primer sets for evaluation the mRNA transcript expressions of spermatogenic genes
